# Neurosurgical videos going mobile

**DOI:** 10.4103/2152-7806.72244

**Published:** 2010-10-30

**Authors:** Pieter L. Kubben

**Affiliations:** Department of Neurosurgery, Maastricht University Medical Center, Netherlands

When we started video implementation for *Surgical Neurology International*, the question was what online video service to choose. Obviously, YouTube (http://youtube.com) is the most well known. It is easy to use, and at that moment it had the best player for mobile devices available. Besides, it was the first video service that seriously started testing with HTML5. This is an upcoming web standard that will play an important role in the future, especially for mobile and tablet devices. But we did not choose YouTube for one reason: the maximum video length is limited to 10 minutes, regardless of the account type you choose.

## VIMEO

After testing another video service first, we found Vimeo (http://vimeo.com) and we liked it from the beginning. It has good video quality, an easy-to-use video player, and includes a full-screen option. Videos can be watched on the Vimeo site, and also integrated in our Web site. Moreover, there are no important restrictions on video length, which makes this service very useful for posting lectures or longer instruction videos.

But until now, there was one restriction in place: videos would only play on desktop or laptop computers, not on mobile devices. We were enthusiastic when Vimeo announced they were making progress with their HTML5 efforts, especially with their mobile video player. And for that reason, we are happy to announce that from this point on, you can watch all of our videos from your mobile device (smartphone, tablet) as well.

## COMMUNITY

The famous Dutch soccer player Johan Cruijff is known for some remarkable statements, and one of them is: “Every disadvantage has its advantage” (or the other way around). To enable mobile video, we needed to redo the video integration in our website, and used that occasion to realize another improvement. Prior to this, you could only place comments or questions to videos on the Vimeo page, but you would have had to create a separate account for that. Not anymore. Now the videos have been integrated in our site in such a way that you can comment directly, in the same manner you can comment on our Posts. We hope that this improved ease of use will stimulate you to engage in our community.

## PODCASTS

With the exception of the UCLA 100 Lectures Series, we are not yet offering podcasts. We did experiment with creating video podcasts, and some technical issues have been solved. At this time, we are ready to create them, but wonder if they have added value next to the mobile video version. The major advantage of a podcast is that you do not need to be online to watch the video once you have downloaded it. This is especially important for developing countries, where an Internet connection is not always available. The major advantage of the standard mobile videos (as available online) is that you can watch them directly without having to download them first. Of course, these two options are not mutually exclusive. Obviously, offering both options will require additional time when preparing the videos.

What is your opinion on podcasts of our videos? Should we be offering them? Would you like to download videos on your mobile device? Or would you only watch them online?

We are looking forward to your opinion, either on our social networks (Twitter, Facebook, LinkedIn) or by E-mail (pieter@kubben.nl).

